# Identification and Phylogenetic Analysis of a Novel Starch Synthase in Maize

**DOI:** 10.3389/fpls.2015.01013

**Published:** 2015-11-20

**Authors:** Hanmei Liu, Guiling Yu, Bin Wei, Yongbin Wang, Junjie Zhang, Yufeng Hu, Yinghong Liu, Guowu Yu, Huaiyu Zhang, Yubi Huang

**Affiliations:** ^1^College of Life Science, Sichuan Agricultural UniversityYa’an, China; ^2^Maize Research Institute, Sichuan Agricultural UniversityChengdu, China; ^3^College of Agronomy, Sichuan Agricultural UniversityChengdu, China

**Keywords:** starch synthase V, maize, starch biosynthesis, phylogenetic analysis, gene duplication

## Abstract

Starch is an important reserve of carbon and energy in plants, providing the majority of calories in the human diet and animal feed. Its synthesis is orchestrated by several key enzymes, and the amount and structure of starch, affecting crop yield and quality, are determined mainly by starch synthase (SS) activity. To date, five SS isoforms, including SSI-IV and Granule Bound Starch Synthase (GBSS) have been identified and their physiological functions have been well characterized. Here, we report the identification of a new SS isoform in maize, designated SSV. By searching sequenced genomes, *SSV* has been found in all green plants with conserved sequences and gene structures. Our phylogenetic analysis based on 780 base pairs has suggested that *SSIV* and *SSV* resulted from a gene duplication event, which may have occurred before the algae formation. An expression profile analysis of *SSV* in maize has indicated that *ZmSSV* is mainly transcribed in the kernel and ear leaf during the grain filling stage, which is partly similar to other SS isoforms. Therefore, it is likely that SSV may play an important role in starch biosynthesis. Subsequent analysis of SSV function may facilitate understanding the mechanism of starch granules formation, number and structure.

## Introduction

Starch, a polymer of glucose, is an important reserve of carbon and energy in plants (Zeeman et al., 2010). In photosynthetic tissues, it is stored as a transient form in chloroplasts during daylight hours and is mobilized at night to provide carbon skeletons and energy for metabolism. In storage tissues, the non-photosynthetic cells use specialized plastids named amyloplasts, which are located in the roots, tubers and seed endosperm, for long-term storage of starch in preparation for future metabolism, i.e., seedling establishment (Brust et al., 2013). Starch in plant storage tissues is the major source of calories in the human diet and animal feed. Furthermore, it is an economical, biodegradable and renewable industrial raw material, widely used in papermaking and first-generation bioethanol production (Smith, 2008; Zeeman et al., 2010).

Starch consists of two classes of α-1,4-glucan polymers, amylose and amylopectin. Amylose is a small linear polymer with few branches, while amylopectin is large and contains frequent α-1,6-branch linkages (Brust et al., 2013). In plants, starch biosynthesis occurs in chloroplasts and amyloplasts and involves a series of biosynthetic enzymes such as ADP-Glcpyrophosphorylase (AGPase) which produces the donor sugar ADP-glucose, SS which uses the ADP-glucose for chain elongation via α-1,4-glycosidic linkages, starch branching enzyme (BE) which creates the α-1,6-linkages, debranching enzyme (DBE) which cleaves the branch to adjust the starch structure, and phosphorylase (Ball and Morell, 2003; Dauvillee et al., 2006; Jeon et al., 2010; Brust et al., 2013). All enzymes act coordinately in the starch biosynthesis processes.

Starch synthases catalyze the transfer of the glucose moiety of ADP-glucose to the non-reducing end of an existing glucan chain via an α-1,4-glucosidic link, which deposits sugars in the starch granules. At least six classes of SSs are recognized in seed plant. To date, five subfamilies including Granule Bound SS (GBSS), SSI, SSII, SSIII, and SSIV have been reported (Yan et al., 2009). Each subfamily has different roles in starch synthesis resulting from their different physicochemical properties and substrate specificities, and has distinct numbers of isoforms in different plants (Ball and Morell, 2003; Jeon et al., 2010). GBSS is responsible for the synthesis of amylose and the extra-long-chain fraction of amylopectin (Wattebled et al., 2002). SSI, SSII and SSIII are involved in the synthesis of amylopectin, elongating the short chains (DP or chain length of 8–12), intermediate chains (DP of 13–25), and long chains (DP of more than 30), respectively (Delvalle et al., 2005; Brust et al., 2013). The function of SSIV has recently been brought to light, controlling the initiation of starch granules (Roldan et al., 2007; Szydlowski et al., 2009). Although the chain-length substrate preference seems to confer non-overlapping function of each SS isoform in starch biosynthesis, research on some *SS* transgenic and mutant lines reveals some redundant function among isoforms (Zhang et al., 2008; Szydlowski et al., 2009). For example, SSIII is responsible for the synthesis of one or two starch granules per chloroplast in single *SSIV* mutant plants. Double mutant *SSIII SSIV* plants do not accumulate starch or any other soluble or insoluble α-linked glucans, indicating the functional redundancy of SSIV and SSIII.

In plants, SSs are GT-B-fold glycosyltransferases, classified within family GT5 in the CAZy database^1^ (Coutinho et al., 2003). The archaeal and bacterial GS are the closest counterparts of plant SSs in the GT5 family (Ball et al., 2011; Cenci et al., 2014), implying that this family is ancient. All of them use ADP-glucose as nucleotide donor sugar. However, GS in other eukaryotes, such as fungi, yeast and animals, are distantly related to plant SSs, and belong to the GT3 family in the CAZy classification, using UDP-glucose as donor. All the SSs in plants and GSs in bacteria share conserved starch catalytic domains (GT5) and glycosyltransferase 1 domains (GT1), including conserved amino acid residues at substrate binding and catalytic sites. The core region, consisting of GT5 and GT1, with basic metabolic activity is encompassed by nearly all of the 60 KDa protein sequence of prokaryotic GSs, corresponding to the C-terminal portion of plant SSs. The N-terminal region of plant SSs, upstream of the core region, is variable with respect to amino acid sequences and lengths among SS isoforms. The N-termini contain a serine-rich region in SSII, three conserved carbohydrate binding modules of family 25 (CBM 25) in SSIII, and two coiled-coil domains in SSIV (Schwarte et al., 2013). The exact function of N-termini is unknown, but may be important in modulating catalytic activity by altering SS kinetics and/or interacting with substrate, or in forming functional enzymatic complexes with other starch biosynthetic enzymes. Although the crystal structures are determined for one archaeal GS, two bacterial GSs and GBSSI in *Oryza sativa* and SSI in *Hordeum vulgare*, the mechanism of glycosyl transfer of the GT5 family remains unclear (Buschiazzo et al., 2004; Sheng et al., 2009; Breton et al., 2012; Momma and Fujimoto, 2012; Cuesta-Seijo et al., 2013).

Five reported subfamilies of SS are well characterized chemically and genetically. Are there any other enzymes involved in plant SBP? What is the characteristic of expression and phylogeny for these novel genes? We have searched sequenced plant genomes to identify novel genes encoding SSs. Interestingly, a putative gene encoding a new SS, named *ZmSSV*, has been located on *Zea mays* chromosome 4 (geneID: GRMZM2G130043). According to the maize genome annotation, *ZmSSV* has two extra long introns, which are intron 4 with a length of 7,398 bp and intron 17 with a length of 54,336 bp. To confirm whether *ZmSSV* could be normally transcribed or is just as a pseudogene, and whether there exist *SSV* homologs in other plants, we have isolated this SS-like gene, which is different from other SS gene subfamilies in maize. Furthermore, the orthologs in some monocots, eudicots, moss and algae have been identified by genome sequence searches. In this paper, the gene structure, expression patterns and evolution of *ZmSSV* are investigated and discussed.

## Materials and Methods

### Plant Material

Maize seedlings (18–599 inbred line, Chinese Elite Corn) were grown in the field at Sichuan Agricultural University Farm in Wenjiang, Sichuan, in April 2014, according to the local standard for high yield maize production. Roots, stems and leaves were taken from 5-leaf-stage seedlings. Silks were harvested before rolling out of the husk, and anthers were harvested during flowering. Developing kernels from self-pollinated ears were collected at 10 DAP, kernels used to separate pericarp, embryo and endosperm were collected at 15 DAP, and ear leaves also collected at 15 DAP. All samples were collected between 9:00 and 10:00 am, and frozen in liquid N_2_ and stored at -80°C until use (Zhang et al., 2014). The sample of 10 DAP kernels was used for isolate the full cDNA of *ZmSSV*, and all the samples were used to the expression analyses.

### RNA Extraction and Full Length cDNA Isolation of *ZmSSV*

Total RNA was extracted from various tissues of maize by Trizol reagent according to the manufacturer’s instructions (Invitrogen^2^). Reverse transcription was carried out by using PrimeScript^TM^ RT reagent Kit (Perfect Real Time) (TaKaRa, Japan), removing contaminating genomic DNA. The forward and reverse primers for the *ZmSSV* full cDNA (∼2.1 kb) isolation were AGGTACGGCGCGCATAGCTAAC and TGTGCTTCTCTAGCAGATGCCCAG, respectively. The PCR was performed using high efficient and fidelity PCR enzyme (KOD FX Neo, Toyobo, Japan) and under the following conditions: 94°C 2 min, 35 cycles of 98°C for 10 s, 60°C for 30 s, 68°C for 150 s, and a final extension at 68°C for 7 min. Amplified PCR products were visualized on a 1% agarose gel and amplicons purified with QIAquick Gel Extraction Kit.

### Expression Analysis by Real-time Quantitative RT-PCR

Primers for qRT-PCR analysis were GAAACTGCTATAGTGGCACCGC (forward) and TCAGGACGATGAAGCTTACGG (reverse), which were specially designed for the length about 200 bp and across an intron. Actin (accession number: NM_001154731.1) was used as the internal control, and its primers were TCACTACGACTGCCGAGCGAG (forward) and GAGCCACCACTGAGGACAACATTAC (reverse). The real-time qRT-PCR was conducted with SYBR Premix Ex TaqTM (Takara) in the Biorad system, according to the manufacturer’s protocols.

### Sequence Retrieval and Analysis

All full-length DNA and amino acid sequences of maize SSs were downloaded from databases of NCBI^3^ and the maize reference genome^4^. In order to retrieve their orthologous sequences in other plants and bacteria, we used all maize sequences as queries against TAIR^5^, NCBI^3^, Phytozome^6^ and Gramene^7^ by performing BLASTP, BLASTN, and TBLASTN programs with *E*-value less than 10^-5^. All the sequences used in the paper were listed in Additional File 1 (**Supplementary Table S1**).

The SSV sequences were aligned by MUSCLE and MAFFT programs, and the alignment result included 645 amino acids. The molecular weights and isoelectric points of deduced protein sequences were predicted by ProtParam^8^, and the signal peptide cleavage sites were predicted with TargetP 1.1^9^. The protein conserved domain prediction was performed using SMART^10^ and CD-search service^11^. Motif analysis was performed by MEME^12^ with the following parameters: repetitions per sequence = zero or one per sequence; maximum number of motifs found = 12; and an ideal motif size between 6 and 300 amino acids (Aoyagi et al., 2014). Jpred^13^ was employed to predict the secondary structure.

### Phylogenetic Analysis

After sequences were aligned and configured for highest accuracy, phylogenetic trees were constructed by multiple methods, including the neighbor-joining, maximum likelihood and maximum parsimony methods, implemented in MEGA, PHYML and PHYLIP on protein sequences of the GT5 domain. Reliability of internal branches was assessed using the bootstrapping method (1000 bootstrap replicates).

## Results

### Cloning and Characterization of *ZmSSV*

Using the maize genome sequence of SS V^4^, we designed primers and isolated the 2190 bp full-length cDNA of *ZmSSV* in maize elite inbred 18–599 (GenBank accession number: KP192927). The amino acid sequence deduced from the open reading frame (ORF) contains 701 residues with a predicted molecular mass of about 78.6 kDa. Our sequencing result is identical to the *ZmSSV* ORF from genome annotation of inbred B73 (Schnable et al., 2009). A chloroplast transit peptide has been predicted in the protein, with a putative cleavage site between amino acid 49 and 50 which would result in a mature protein with calculated molecular mass of 73.4 kDa. It is likely that ZmSSV is localized within the chloroplast, similar to other SSs such as SSI and SSIV (Delvalle et al., 2005; Roldan et al., 2007).

**FIGURE 1:**
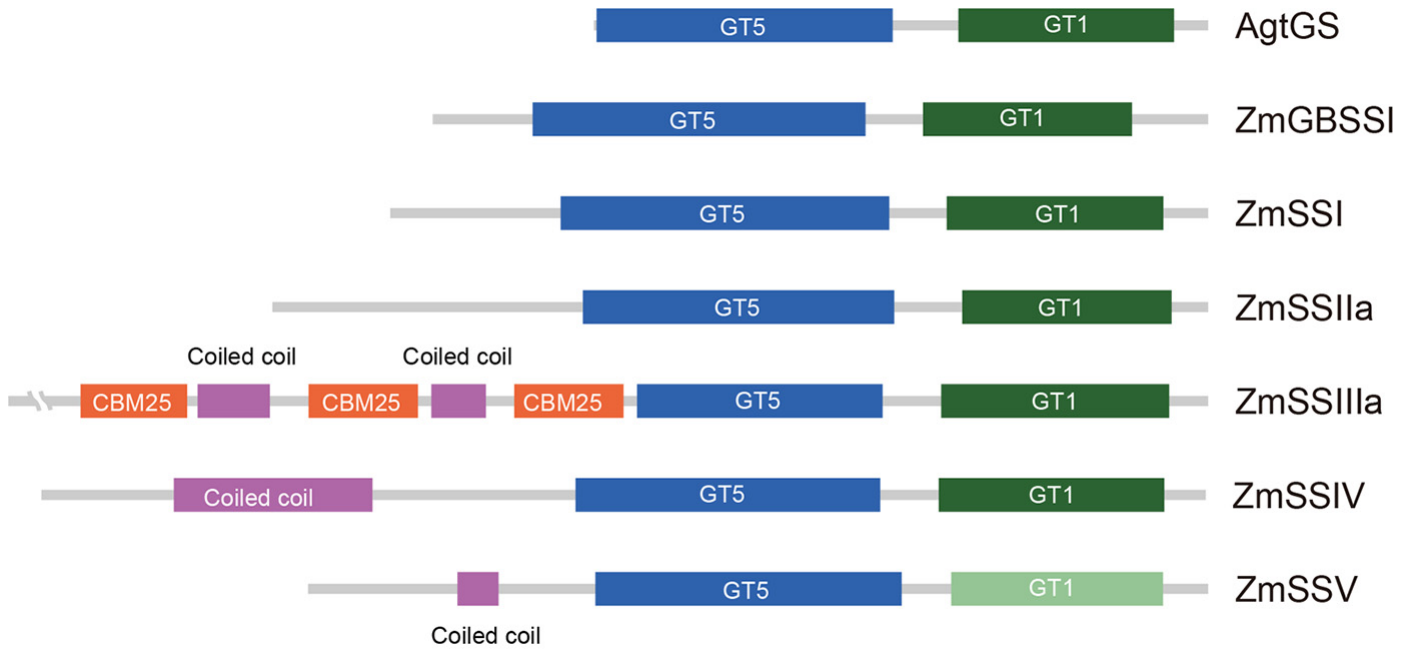
**Domain structure of the six starch synthase proteins from maize.** For comparison, the glycogen synthase of *Agrobacterium tumefaciens* (AgtGS) was involved. The N-terminus of enzymes was at the left, and the C-terminus was at the right. The conserved domains of the glycosyltransferase family 5 (GT5) and the glycosyltransferase family 1 (GT1) were showed in blue and green, respectively. Because of the failure prediction GT1 in ZmSSV by SMART, the conserved equivalents of sequence alignment were given in light green as the putative GT1-like domain. The N-terminus of ZmSSIII contained three conserved carbohydrate binding modules of family 25 (CBM 25) marked in red, and coiled coil domains in ZmSSIII-V were marked in pink.

The putative amino acid sequence of ZmSSV is most closely related to ZmSSIV. Sequence alignment shows 35% identity, with consensus sequences not only in the conserved C-terminus including starch catalytic and glycosyltransferase domains, but also in the unique N-terminus. The GT5 domain is more conservative than GT1 in plant SSs (Leterrier et al., 2008). In ZmSSV, the GT5 domain could be predicted with low *E*-value of 1.7e-32 by SMART and CD-search programs, and prediction of the GT1 domain failed. However, sequence identity of the GT1 domain among SS subfamilies in maize and between other species is obviously higher than those of non-domain regions. We have designated it a putative GT1-like domain, shown in light green in **Figure 1**. The length and relative positions of GT5 and GT1 domains in ZmSSV are similar to those of GBSS and SSI-IV in maize and the GS in *Agrobacterium tumefaciens* (AgtGS) (**Figure 1**). Besides the GT5 and GT1 domains, there is a coiled coli domain between amino acids 117 and 147 in the ZmSSV N-terminus, similar to the coiled coli domain of ZmSSIV but absent from other SS subfamilies. Moreover, the sequence identity of the coiled coil domain between ZmSSV and ZmSSIV is significantly higher than that of the surrounding region, and there are some conserved sites (**Supplementary Figure S1A** in Additional File 2). We infer that the two domains may perform similar or identical functions. With these conserved domains, ZmSSV is identified as a SS.

### Conserved Genomic Structure of *SSV* in Maize and Other Angiosperm

**FIGURE 2:**
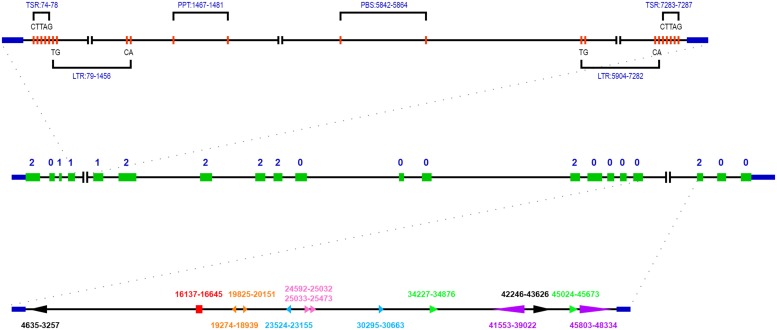
**Structure organization of *ZmSSV* gene according to the B73 reference genome.** The solid green boxes indicated exons, and the number of 0, 1, and 2 represented intron phase. The specific structures and sequence features of extra long intron 4 and 17 were marked. In intron 4, the position of two long terminal repeats (LTR), a primer binding sites (PBS) and a polypurine tract (PPT) were showed, and the bases of LTR end and the target site duplication were indicated too. In intron 17, a pair of arrows with the same color and shape represented a pair of repeat segments, arrows pointing in the same direction indicated direct repeat, pointing in the opposite direction indicated inverted repeat. Arrows with the same direction and consecutive position indicated tandem repeat. Red rectangular indicated that some short repeat segments were included in the region.

The genomic sequence of *ZmSSV* is about 70.9 kb in the B73 reference genome (Schnable et al., 2009). We have aligned the genomic DNA sequences with full-length cDNA and revealed that *ZmSSV* contains 20 exons separated by 19 introns, of lengths shown in **Table 1** and **Figure 2**. Two introns, 4 and 17, are remarkably long. Assuming that the B73 reference genome of the region has been sequenced and assembled accurately, *ZmSSV* is much larger than the average maize gene (Schnable et al., 2009).

**Table 1:** Sizes of exons and introns in the *SSV* gene.

Exon^∗^	Maize	Sorghum	Rice	Soybean	Potato	Poplar	*Arabidopsis*^∗∗^	Moss	Algae	Intron	Maize	Sorghum	Rice	Soybean	Potato	Poplar	*Arabidopsis*	Moss	Algae
1	170	170	164	95	113	122	116	128	35	1	141	118	138	229	318	79	234	259	181
2	57	57	57	36	48	107	34	34	106	2	95	79	99	93	109	70	73	396	169
3	28	28	28	34	25	0	0	341	246	3	101	99	110	124	92	0	0	66	190
4	67	58	70	70	55	0	79	200	103	4	7349	172	181	82	95	0	92	445	151
5	114	114	114	129	129	117	129	0	142	5	220	220	204	850	833	96	111	0	119
6	223	223	223	205	205	208	205	256	148	6	874	6790	301	361	462	667	86	380	147
7	135	135	135	135	135	135	135	135	84	7	608	628	328	99	488	777	81	133	236
8	105	105	105	105	105	105	105	105	159	8	130	132	226	184	221	182	136	188	110
9	96	96	96	99	99	99	99	99	147	9	200	202	385	973	1760	1148	91	111	102
10	130	130	130	130	130	130	130	130	321	10	1253	993	416	726	924	773	77	523	130
11	51	51	51	51	51	51	48	51	156	11	254	248	554	174	99	95	97	336	115
12	111	111	111	111	111	111	111	111	176	12	1882	403	618	576	338	1415	73	147	162
13	110	110	110	110	110	110	89	110	115	13	121	123	113	88	187	190	79	285	
14	166	166	166	169	166	169	103	172		14	87	87	84	993	680	1522		301	
15	81	81	81	87	87	84	N/A	87		15	92	92	86	295	357	241		264	
16	69	69	69	78	72	69	N/A	66		16	106	105	123	451	81	449		108	
17	114	114	114	114	114	114	N/A	114		17	54336	495	486	331	741	573		171	
18	62	62	62	62	62	62	N/A	62		18	210	199	133	80	102	111		143	
19	97	97	97	115	97	97	N/A	115		19	213	214	161	286	179	86		221	
20	120	120	120	120	120	120	N/A	120											


*^∗^The first and last exons were represented as the encoding sequence. ^∗∗^N/A indicated not available, maybe because the genomic sequences covering the region had not been completely sequenced.*

Although *SSV* homologs in several angiosperm have been annotated, some putative ORFs and proteins are much shorter than that of maize, such as in *Sorghum bicolor* and *Arabidopsis thaliana*, suggesting that these annotations may not be accurate. To identify the complete SSV gene in plants, we performed BLAST analysis in NCBI using the *ZmSSV* CDS and protein as query. We identified the complete *SSV* CDS of *O. sativa* (Japonica Group) (EU621837) and *Solanum tuberosum* (EU661369), then aligned to the corresponding genome sequences to calculate exon lengths and determine the introns according to the “GT(C)-AG” rule (Breathnach and Chambon, 1981). Similarly, we have aligned *ZmSSV* CDS to the sorghum genome, designed primers and isolated the complete sorghum *SSV* gene (*SbSSV*, GenBank accession number: KP192926). We found no full length *Arabidopsis SSV* CDS in GenBank, and were not successful in aligning the homologous *SSV* CDS of other eudicots with the *Arabidopsis* genome, so could not get the complete *AtSSV* ORFs.

The exon and intron structure of *SSV* is generally conserved in land plants (**Table 1**), suggesting ancient common ancestry of these genes. Of the 20 exons in monocots, 18 are equal in length, only the first and fourth varying slightly. In eudicots, *SSV* of *Glycine max* (soybean) and potato have 20 exons, and *Populus trichocarpa* has 18. *Arabidopsis SSV* has only 13 exons and 460 putative amino acids, suggesting that the genome annotation of *AtSSV* is not complete. Between soybean and potato, 14 of 20 exons are equal in length, 11 of these also being equal to poplar exons, and five to *Arabidopsis* exons (despite the incomplete annotation of *AtSSV)*. Between monocots and dicots (excluding *Arabidopsis*), nine *SSV* exons are equal in length, also being equal to those of moss (*Physcomitrella patens*). SSV structure in algae (*Chlamydomonas reinhardtii*) is substantially different from land plants, suggesting rapid evolution of the gene in algae after divergence from a common ancestor shared with land plants.

The length of introns in *SSV* varies widely among species, due to lineage specific events. In maize, for example, a transposable element (TE) belonging to the long terminal repeat retrotransposon copia class with length of 7204 bp locates in intron 4, and a number of variable repeat sequences locate in intron 17 (**Figure 2**). Using LTR_FINDER (Xu and Wang, 2007), we found that the TE locates between positions 79 and 7282 with typical characteristics. In intron 17, there are many repeated segments with variable lengths from 2522 to dozens of bp. Some repeated segment pairs are direct, some inverted and some tandem. The long repeated segment pairs are shown in **Figure 2** with the same marks. Like maize, there are also large introns in sorghum, potato and poplar. These large introns do not correspond to one another, so it is inferred that lineage specific events such as insertion of a maize LTR retrotransposon (above) have affected them.

### Phylogenetic Relationships between SSV and Other Starch Synthases

**FIGURE 3:**
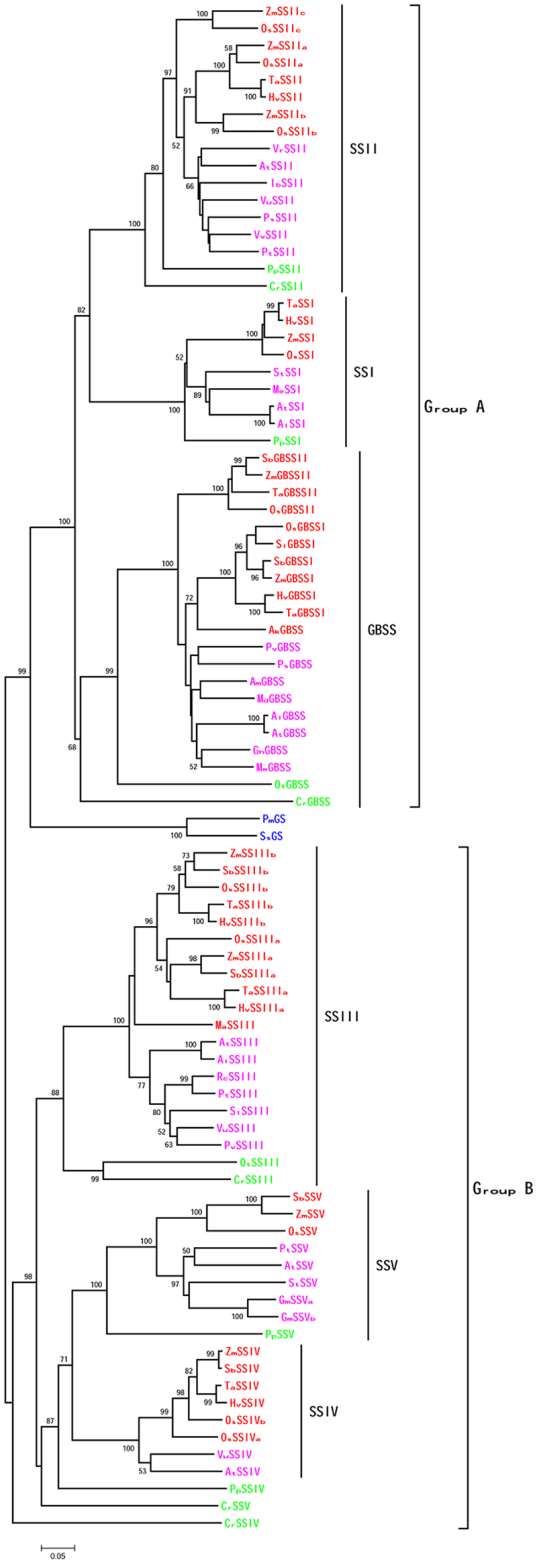
**Phylogenetic tree showing the relationship among plant SSs.** The tree was constructed using the neighbor-joining method, and numbers on branches represented bootstrap estimates for 1000 replicate analyses, only bootstrap scores higher than 50 were shown. Each node was labeled with the prefix of the initials of the genus and species. Monocots were showed in red, eudicots in pink, algae and moss in green, and bacteria in blue.

To investigate the relationship between SSV and other SSs, we collected SSs from more than 20 plant species (including monocots, eudicots and other chlorobiontes) and GSs from two bacteria (*Prochlorococcus marinus AS9601* and *Synechoccus sp. CC9311*). The topologies of phylogenetic trees, constructed by multiple methods, mostly agree with one another. The six SS classes cluster into two groups, each including three clades (**Figure 3**). For ease of description, we have designated GBSSI, SSI and SSII as group A, and SSIII, SSIV and SSV as group B. Within Group B, SSV of all plants except algae forms a new clade, separate from SSIII and SSIV. The phylogenetic relationship between SSIV and SSV is closest, perhaps suggesting that they result from an ancient gene duplication, consistent with the generation of other SS subfamilies. The phylogenetic relationships among GBSS and SSI-IV are consistent with previous reports (Leterrier et al., 2008; Nougue et al., 2014).

Monocots and eudicots form two sub-branches of the SSV clade, with SSV of moss as out-orthologues, consistent with the species phylogeny. The PpSSIV and CrSSV form two branches outside of the SSIV and SSV clades, and CrSSIV is outgroup to all of Group B, supported with high bootstrap values, perhaps reflecting the rapid evolution of these genes in lower plants (Cenci et al., 2014).

### Comparison of SSV Features to those of Other Starch Synthases

**FIGURE 4:**
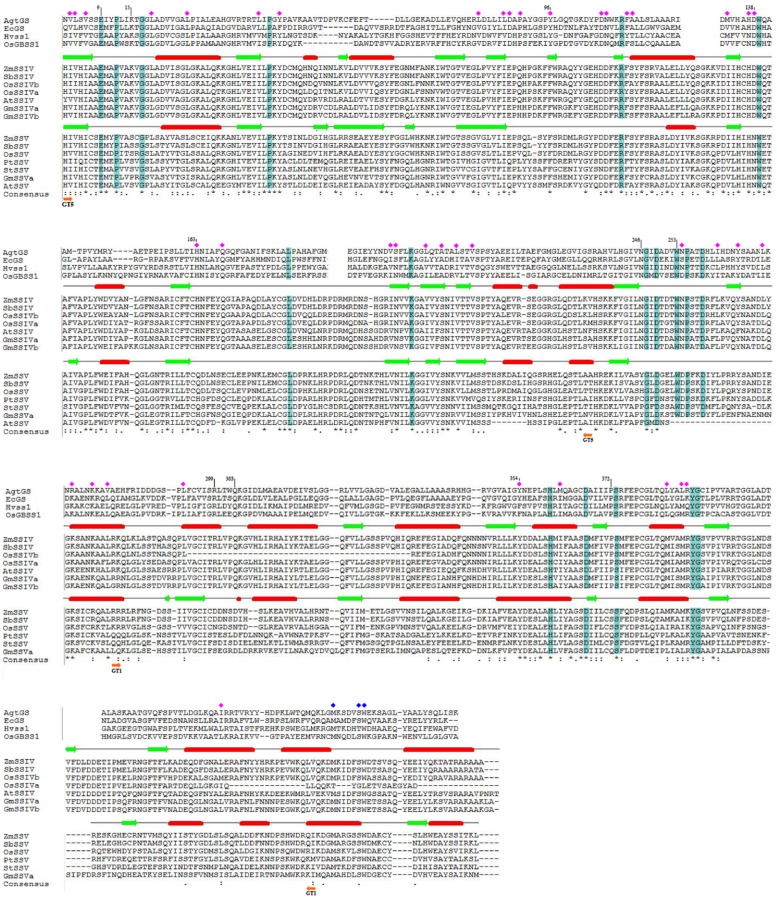
**The protein sequence alignment of SSIV and SSV in eudicots and monocots, for C-terminus conserved region.** For further investigation the important functional sites, partial alignment with GSs and SSs of which the 3D structure were determined were showed. Moreover, the secondary structure of ZmSSIV and ZmSSV were predicted, α-helical indicated by red cylinder, and β-strand indicated by green arrows. The identical amino acid residues in a colum were highlighted in blue, or noted by blue squares(except the differing residues of OsSSIVa end), and conserved amino acid residues were noted by pink squares. In the line of the consensus, identical and conserved residues between SSIV and SSV were marked with asterisks (^∗^) and dots/colons (./:), respectively, while, dashes indicate no residue. The boundary regions of GT5 and GT1 were marked with orange arrows.

SSV should share with other SSs features responsible for the conserved functions of SSs, and have unique features responsible for its specific functions. To further investigate SSV features, we aligned the SSIV and SSV protein sequences of monocots and eudicots. In the variable N-terminus, both the coiled coil domain and the other conservative region adjoining the GT5 domain had multiple identical and conserved amino acid sites (**Supplementary Figure S1B** in Additional File 2). For the C-terminus composed of the conserved starch catalytic (GT5) and glycosyltransferase-1 (GT1) domains, we aligned SSV not only with SSIV, but also with the glycogen synthase of *A. tumefaciens* and *Escherichia coli*, and rice GBSSI and barley SSI, for which the crystal structures have been determined (Buschiazzo et al., 2004; Sheng et al., 2009; Momma and Fujimoto, 2012; Cuesta-Seijo et al., 2013) (**Figure 4**). The consensus lines, last line of the alignment, composing of asterisks (*) and dots/colons (./:), have revealed identical and conserved residues of SSIV and SSV. Overall sequence identity is high, especially in the GT5 domain, with that of the GT1 domain slightly lower. One of the possibilities for lower sequence identity in the GT1 region between SSIV and SSV, is the rapid evolution accompanying functional divergence of SSV after gene duplication. Another possibility is that some members of SSIV, such as OsSSIVa and OsSSIVb, accumulate more deletion mutations. As the incomplete sequence of SSV in *Arabidopsis*, AtSSV is not involved in the alignment of GT1 region.

Because of the distant phylogenetic relationship between GSs and SSs in plant, we just showed partial alignment of conserved regions between AgtGS, EcGS, OsGBSSI, and HvSSI, and SSIV–V in **Figure 4**. Identical residues in a column are highlighted in blue, and conserved residues (with similar physicochemical properties) are noted by pink squares. We have listed the important functional residues (**Table 2**) to directly bind and catalyze the substrate in AgtGS, EcGS, OsGBSSI, and HvSSI as reported previously (Buschiazzo et al., 2004; Sheng et al., 2009; Momma and Fujimoto, 2012; Cuesta-Seijo et al., 2013), and identified the equivalent residues in ZmSSIV and ZmSSV by the protein sequence alignment. The corresponding positions of these functional sites in AgtGS are marked in **Figure 4**. Although the phylogenetic relationship among SSIV-V, GSs, OsGBSSI and HvSSI is variable, most of the amino acid residues of these functional sites are identical or conservative in all enzymes. There are eight identical and 27 conserved sites in GT5; and five identical and seven conserved sites in GT1. Interestingly, amino acid residues of the region between GT5 and GT1 are very conservative too. The length of the region is about 30 amino acid residues, which includes five identical and seven conserved sites. Moreover, three reported functional sites in AgtGS (N246, G247, W253) locate in this region. Likewise, in the adjoining region of the GT1 end, there are several identical and conserved residues (marked with blue squares in **Figure 4**) in all GSs and SSs, excepting the OsSSIVa end with great divergence. Comparing the conserved functional sites in SSV with other plant SSs and bacterial GSs (**Table 2**), more amino acid variants are found in SSV. For example, K15 in AgtGS is replaced by S237 in ZmSSV, and H163 in AgtGS is replaced by Q387, both substitutions from basic residues to uncharged amino acids, which would likely affect the functional properties of the enzymes. SSV may have significant functional divergence from other SSs.

**Table 2:** Amino acid residues of substrate binding and/or catalyzing sites in structure available GSs and SSs, and their corresponding residues in ZmSSIV and ZmSSV.

Enzymes	Substrate binding and/or catalyzing sites																					
AgtGS	9	**E**	15–22	KT**G**G**L**ADV	96	Y	138–140	D**W**Q	163–164	HN	246–247	N**G**	253	**W**	299–300	RL	303–305	QKG	354	Y	372–383	PSRFEPCGLTQL
EcGS	9	**E**	15–22	KT**G**G**L**ADV	95	Y	137–139	D**W**H	161–162	HN	246–247	N**G**	253	**W**	300–301	RL	304–306	QKG	355	Y	373–384	PSRFEPCGLTQL
OsGBSSI	91	**E**	97–104	KT**G**G**L**GDV	183	Y	234–236	D**W**H	264–265	HN	353–354	N**G**	360	**W**	408–409	RL	412–414	QKG	463	F	481–492	PSRFEPCGLIQL
HvSSI	143	**E**	149–156	KS**G**G**L**GDV	235	Y	280–282	D**W**H	310–311	HN	404–405	N**G**	411	**W**	458–459	RL	462–464	QKG	513	F	531–542	PSRFEPCGLNQL
ZmSSIV	424	**E**	430–437	KV**G**G**L**ADV	512	F	552–554	D**W**Q	581–582	HN	666–667	N**G**	673	**W**	721–722	RL	725–727	QKG	779	Y	797–808	PSMFEPCGLTQM
ZmSSV	231	**E**	237–244	SC**G**P**L**SAY	318	F	358–360	N**W**E	387–388	QD	474–475	Y**G**	481	**W**	527–528	DD	531	D—-	581	Y	599–610	SSFQDPSLQIAM


*Amino acids that were part of the substrate binding and/or catalytic sites in AgtGS, EcGS, OsGBSSI and HvSSI were identified in previous studies (Buschiazzo et al., 2004; Sheng et al., 2009; Momma and Fujimoto, 2012; Cuesta-Seijo et al., 2013), and the equivalent residues in ZmSSIV and ZmSSV were identified after alignment using mafft. Data was extracted from the alignment shown in **Figure 4**. The identical residues in all GSs and SSs were in bold.*

To identify the specific structural features of SSV, the secondary structures of ZmSSIV and ZmSSV (**Figure 4**) have been predicted using the Jpred program. The greatest divergence of secondary structure between ZmSSIV and ZmSSV is in the GT5 domain. One α-helix and two β-strands are absent in ZmSSV, and one α-helix of ZmSSIV changes to β-strand in the corresponding region of ZmSSV. In the starting region of GT1, one α-helix of ZmSSIV also changes to β-strand in ZmSSV. Two additional β-strands, located in GT1, and at the end of the C-terminus, respectively, are found only in ZmSSV. Outside of these regions, the secondary structure of the two proteins is quite conservative.

**FIGURE 5:**
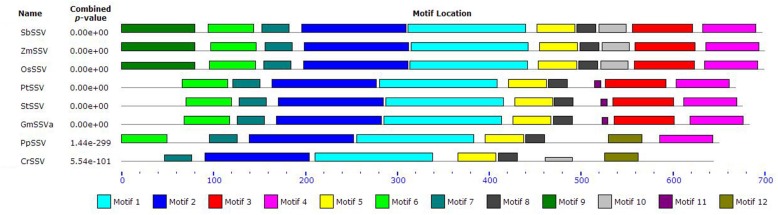
**The conserved motifs of SSV**.

The alignment also indicates some amino acid residues that vary within the SSV subfamily. To further reveal the potential functional regions or divergent sites in SSV, we have analyzed conserved motifs of SSV members by the MEME program, finding that there exist different motifs in SSV from algae to monocots (**Figure 5**). Five motifs (1, 2, 5, 7, and 8) are found in all members. Motif 9 and 10 are only found in monocots, and motif 11 is only in eudicots. Motifs in PpSSV and CrSSV are different from those of angiosperm, lacking motifs 3, 10, and 11 but with a unique motif (12). The results of motif investigation are consistent with the SSV gene phylogeny, indicating that each branch of SSV has unique functional regions and sites.

### Expression Patterns of *ZmSSV*

**FIGURE 6:**
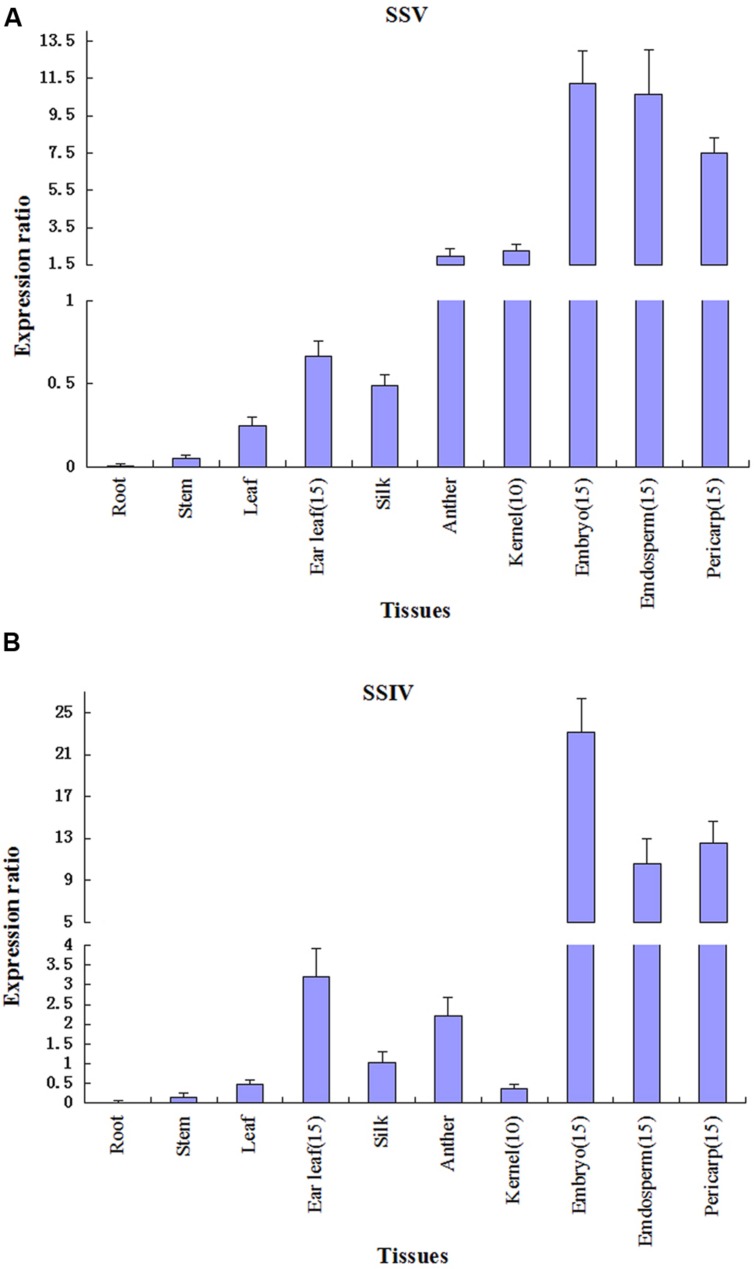
**Quantitative RT-PCR analysis of *ZmSSIV***(B)** and *ZmSSV***(A)** in different tissues.** Gene expression was normalized to actin for each sample. Values are averages from three independent biological experiments ± SE.

Quantitative RT-PCR analysis has been performed to study the expression profiles of transcripts encoded by *SSV* in maize, and the result is reproducible over three different experiments. We focus on the expression level of *ZmSSV* in various tissues at vegetative and reproductive stages. The *ZmSSV* transcript is very weakly expressed in root and stem, with slightly higher expression in leaf of 5-leaf-stage seedlings, but is mainly expressed in male and female floral organs, especially in the ear (**Figure 6A**). The level of *ZmSSV* transcription in anther is higher than that of silk. The highest expression level is in 15 DAP embryo and endosperm, followed by 15 DAP pericarp. Furthermore, because the 10 DAP kernel is too small to effectively partition the pericarp, endosperm and embryo, we tested the whole kernel, finding strong *ZmSSV* expression that was obviously higher than in vegetable tissues, but lower than in the 15 DAP kernel. The ear leaf, one of the vegetative tissues, is an important site of sucrose synthesis and supply to grain filling. *ZmSSV* is expressed highly in the ear leaf at the grain filling stage of 15 DAP, and its level is significantly higher than in seedling leaves.

Expression of *ZmSSIV* is compared to that of *ZmSSV*, because of the close phylogenetic relationship between the two isoforms (**Figure 6B**). The highest *ZmSSIV* expression is also in the embryo, endosperm and pericarp at 15 DAP, and then in ear leaf, anther and silk. The *ZmSSIV* expression level is not high in 10 DAP kernels, and the lowest level is in the seedling root, stem and leaf. So the expression profiles across tissues of *ZmSSIV* and *ZmSSV* are similar. In addition, similar expression profiles have been found in homologues of *ZmGBSSI*, *ZmSSI*, *OsSSIII-2* and *OsSSIV-1* and others (Dian et al., 2005; Leterrier et al., 2008; Chen et al., 2011). In conclusion, *ZmSSV* mainly expresses in the ear, and its transcript reaches its highest levels in the grain filling stage, suggesting that *ZmSSV* may be very important to starch synthesis in grain.

## Discussion

Starch synthases are enzymes essential to produce starch, a semicrystalline-storage polysaccharide in plants. Previously, five subfamilies of GBSS and SSI-SSIV have been isolated in plants and physiological functions revealed (Brust et al., 2013; Nougue et al., 2014). Here, we report a new SS-like gene subfamily, SSV, with the GT5 and GT1 domains shared by all plant SSs and bacterial GSs. Just as with other SSs, SSV is identifiable based on conserved sequence and gene structure from lower to higher green plants. SSV is much more related to SSIV than to other SSs, according to phylogenetic analysis and sequence alignment. It is thus concluded that SSV is a new SS subfamily, and the number of SS isoforms is now at least six in plants.

The SBP is a complex network of genes, most of which are members of large multigene families with multiple isoforms. For these genes, only a few mutations have resulted in readily observed morphological phenotypes, such as the well known endosperm mutants of *ae*, *sh1*, *sh2*, *du*, and *wx* in maize (Gao et al., 1998; Ball and Morell, 2003; Crofts et al., 2012; Sparla et al., 2014). The majority of gene mutations alter the properties of starch, but have small effects on starch yield, leading to little or no morphological phenotype. Furthermore, as the analysis of amylose and amylopectin structure is highly complex, phenotype-based cloning of mutant starch biosynthesis genes are of limited scope. To date, two approaches, homology hybridization screening or PCR screening based on conserved known genes sequence, and protein isolation, purification and sequencing are the most widely used strategies for cloning starch biosynthesis genes (Zeeman et al., 2010). Each approach has limitations. Homology-based searches may fail to identify new families that have low sequence similarities with known families. Protein isolation may fail to purify all isozymes, as different isozymes may have extremely similar activity and molecular weight in plants (Brust et al., 2013). Recently, databases of whole genomes together with full-length cDNAs in many plant species have provided unique opportunities to comprehensively study SS families. Combination of DNA sequence information with molecular biology experiments is an effective mean for new gene isolation.

Starch is found mostly in lineages derived from primary plastid endosymbiosis, the Archaeplastida, which involves the Chloroplastida (green algae and land-plants), the Rhodophyceae (red algae), and the Glaucophyta (glaucophytes) (Ball et al., 2011; Cenci et al., 2014). Starch is also found in some unicellular marine diazotrophic cyanobacteria and several secondary endosymbiotic lineages, which suggests that the SBP existed in the cyanobiont before endosymbiosis (Deschamps et al., 2008a,b). In the Archaeplastida, starch synthesis has been found in the cytoplasm of Rhodophyceae and Glaucophyta, but is localized to the chloroplast stroma of Chloroplastida. Investigation of the phylogenetic origin of genes in the Chloroplastida SBP showed that some enzymes originate from cyanobacteria, and others originate from eukaryotic hosts (Deschamps et al., 2008b). Dauvillee et al. (2006), Deschamps et al. (2008a,c), Colleoni et al. (2010), Ball et al. (2011), and Cenci et al. (2014) have researched the evolution of starch metabolism in plants, and put forward a reasonable interpretation of SBP gene origin. It suggested that an ancestor of present day cyanobacteria was internalized, probably through phagocytosis by a heterotrophic eukaryotic cell, then the export of photosynthate from the cyanobiont to the host cytosol provided the eukaryotic world with the ability to perform oxygenic photosynthesis. As the cyanobiont slowly became a true organelle, the majority of cyanobacterial genes were lost as they were neither involved in oxygenic photosynthesis nor essential for maintenance and division of the symbiont. During this process, some cyanobacterial SBP genes were transferred into and retained by the host nucleus and immediately expressed in the cytosol for starch synthesis. Subsequently, the novel light-harvesting antennae appeared in chloroplasts, which defined the most distinctive feature of the Chloroplastida. To coordinate the metabolic balance of the Chloroplastida light-harvesting complexes in cells, such as maintaining the ATP charge in darkness and obviating oxidative stress, the starch metabolism pathway was redirected to chloroplasts (Deschamps et al., 2008a,b,c). Three eukaryotic lineages of the Chloroplastida, the Rhodophyceae and the Glaucophyta emerged after or during the metabolic integration of the plastid. With the progressive evolution of coordinated and complex starch metabolism in the cytosol and chloroplast of photosynthetic tissues, and the cytosol and amyloplasts of storage tissues for seed plant, the number of genes involved in SBP has apparently increased, largely due to gene duplication and accompanying functional divergence, resulting in multiple enzyme isoforms (Deschamps et al., 2008c; Nougue et al., 2014). These isoforms play only partly redundant functions and are often responsible for distinctive roles in the starch formation of different substructures.

In this paper, phylogenetic analysis has suggested that SS isoforms of GBSS and SSI-V are present in all green plants (from algae to monocots). We infer that these isoforms have originated from early gene duplications, which may have occurred in the Archaeplastida ancestor or during the process of SBP redirection to the chloroplast (Nougue et al., 2014). In Group A, the first duplication led to paralogues encoding GBSS and the ancestor of SSI and SSII, and the second duplication produced the SSI and SSII isoforms. The branch of two cyanobacterial GSs is located outside of Group A with 100% bootstrap values. It was inferred that the ancestors of GBSS and SSI-II were acquired through endosymbiotic gene transfer from a plastid ancestor, with cyanobacterial starch synthesis genes transferred initially to host cytosol, and subsequently relocated to the plastid (Deschamps et al., 2008b). The two gene duplications may have occurred during this latter step, of relocating starch synthesis to the plastid. In Group B, two duplications also led to three paralogous clades encoding SSIII, SSIV, and SSV. However, the origin of the ancestor of SSIII-V was different from Group A, thought to have been transmitted from intracellular chlamydiae pathogens, more recently than the transfer of the GBSS ancestor (Nougue et al., 2014). However, Leterrier et al. (2008) analyzed two forms of GS in *Synechocystis* PCC 6803, and found that *Sp*GS2 was closely related to the GBSSs, while *Sp*GS1 was more similar to SSIV. They suggested that the Group A and Group B SSs evolved directly from the two independent GS types, the SSIV group evolved from *Sp*GS1, and subsequent gene duplication events might lead to the evolution of SSIIIs. The exact origin of the ancestor of Group B SSs has not been determined, and further investigation is needed. The duplication leading to the evolution of SSIII must have been earlier than the duplication that we infer to have produced closely related SSIV and SSV, the latter perhaps occurring before or during the green plant lineage divergence.

To further investigate the structure-function relationship of SSs, we have built a homology model for the C-terminus of ZmSSIV and ZmSSV (data not shown) based on the HvSSI structure using Swiss-model^14^. Just as is found in the structures of HvSSI, OsGBSSI and two GSs, the two Rossmann-fold like α-β-α domains are apparent in ZmSSIV and ZmSSV, and the large cleft separating both domains makes up the substrate-binding and catalytic center. Two classes of functional sites exist in the large cleft. One class is the ADP-binding pocket, which consists of the C-terminal domain wall and the conserved N-terminal KTGGL loop motif. The exact amino acid residues of the ADP-binding pocket in AtgGS include KTGGL(15–19), R299, Y354, E376 and T381 (Buschiazzo et al., 2004). Comparing these residues among SSs isoforms (**Figure 4**; **Table 2**), several amino acid replacements with different characteristics have been found in SSV, such as K15/S, G18/P, R299/D and T381/I. The other class of functional sites are the acceptor (starch or maltooligosaccharide) binding and catalyzing sites, including E9, Y96, DWQ(138–140) in AtgGS, and others. Some of the maltooligosaccharide-binding sites are conserved, but parts are flexible. Changes of non-conservative amino acids could affect enzyme properties. For example, Cuesta-Seijo et al. (2013) tested mutant HvSSI_F538A (the important surface maltooligosaccharide binding site) with different substrates, including maltopentaose, glycogen and soluble starch. Compared with the wild-type enzyme, the activity of the F538A mutant is 12-fold lower with rabbit liver glycogen, sixfold lower with oyster glycogen and fourfold lower with soluble starch at high glycogen or starch concentrations. The substrate binding and/or catalyzing sites of RL(299,300) and QKG(303-305) in AgtGS were substituted for residues with different charge and deleted in ZmSSV. Moreover, dozens of amino acids in the counterpart region were deleted in OsSSIVb (**Figure 4**; **Table 2**), suggesting that these sites in SSs were flexible in different plant species. Collectively, substitutions in key amino acid residues and differences in secondary structure near or at the active/binding sites may lead to different spatio-conformational outcomes and possibly change interaction of protein with substrate. In this paper, we have found amino acids in substrate-binding and catalytic centers that are distinct between SS isoforms, which may partially explain important functional differences of each SS isoform. The significant difference of key amino acid residues between SSV and other isoforms has suggested that SSV may play roles in starch synthesis, that are distinct from the known isoforms of GBSS and SSI–SSIV.

SSV shows more relatedness to SSIV than to other SSs. The specific function of SSIV is in the control of starch granule formation. SSIV defective mutants of *Arabidopsis* display a severe growth defect, and the number of starch granules per plastid is dramatically decreased, leading to a single large particle per plastid, but the starch content and structure is near that of wild-type plants (Roldan et al., 2007; Gamez-Arjona et al., 2011). Further analysis has suggested that the role of SSIV in granule seeding could be replaced, in part, by the phylogenetically related SSIII, which causes no readily observed morphological change in the SSIV mutants (Szydlowski et al., 2009). We infer that the physiological function of SSV may be similar to those of paralogues of SSIII and SSIV, and these isoforms produced by gene duplication may have partly redundant functions. Expression profile analysis has showed that SSV is mainly expressed in the tissues of ear leaves and kernels, where transient and storage starch are synthesized, respectively. It is inferred that SSV may play some role(s) in starch synthesis. Additional studies will be conducted to decipher the function of the new SSV subfamily by analysis of double and/or triple mutants of SSIII–V. If we were to assume that SSV did not play any roles, it would be quite puzzling as to why these genes have been retained in all green plants, and are so highly conserved in their structural features and protein sequences.

## Conclusion

In summary, we have cloned the full CDS of *ZmSSV*, then used it as query sequence to identify *SSV* in other green plants (from algae to monocots) by aligning to genome sequences. Conserved sequences and gene structures of *SSV* orthologs indicate that *SSV* in plants must be derived from the same ancestor. *SSV* is most related to the known *SSIV*, and they are produced by a gene duplication occurring in the early stages of the evolution of green plants. Although molecular evolution has conferred functional divergence of the duplicated gene pairs, their expression profiles remain similar.

## Author Contributions

YH, HL, and HZ conceived and designed the experiments. YL contributed plant materials. HL, BW, and GY contributed to data analysis. GY, YH, JZ, YW, and GY performed the experiments. HL wrote the manuscript. All authors discussed the results and commented on the manuscript.

## Conflict of Interest Statement

The authors declare that the research was conducted in the absence of any commercial or financial relationships that could be construed as a potential conflict of interest.

## Footnotes

1 http://www.cazy.org

2 http://www.invitrogen.com

3 http://www.ncbi.nlm.nih.gov/

4 http://ensembl.gramene.org/Zea_mays/Info/Index

5 http://arabidopsis.org/

6 http://www.phytozome.net

7 http://www.gramene.org/

8 http://web.expasy.org/protparam/

9 http://www.cbs.dtu.dk/services/TargetP/

10 http://smart.embl-heidelberg.de/

11 http://www.ncbi.nlm.nih.gov/Structure/cdd/wrpsb.cgi

12 http://meme.nbcr.net/meme/cgi-bin/meme.cgi

13 http://www.compbio.dundee.ac.uk/www-jpred/

14 http://swissmodel.expasy.org/

## ABBREVIATIONS

DAP: days after pollination
DP: degree of polymerization
GBSS: Granule Bound starch synthase
GS: glycogen synthases
SBP: starch biosynthesis pathway
SS: starch synthase.

## References

[B1] Aoyagi L. N. Lopes-Caitar V. S. De Carvalho M. C. Darben L. M. Polizel-Podanosqui A. Kuwahara M. K. (2014). Genomic and transcriptomic characterization of the transcription factor family R2R3-MYB in soybean and its involvement in the resistance responses to *Phakopsora pachyrhizi*. *Plant Sci.* 229 32–42. 10.1016/j.plantsci.2014.08.005 25443831

[B2] Ball S. Colleoni C. Cenci U. Raj J. N. Tirtiaux C. (2011). The evolution of glycogen and starch metabolism in eukaryotes gives molecular clues to understand the establishment of plastid endosymbiosis. *J. Exp. Bot.* 62 1775–1801. 10.1093/jxb/erq411 21220783

[B3] Ball S. G. Morell M. K. (2003). From bacterial glycogen to starch: understanding the biogenesis of the plant starch granule. *Annu. Rev. Plant Biol.* 54 207–233. 10.1146/annurev.arplant.54.031902.134927 14502990

[B4] Breathnach R. Chambon P. (1981). Organization and expression of eucaryotic split genes coding for proteins. *Annu. Rev. Biochem.* 50 349–383. 10.1146/annurev.bi.50.070181.002025 6791577

[B5] Breton C. Fournel-Gigleux S. Palcic M. M. (2012). Recent structures, evolution and mechanisms of glycosyltransferases. *Curr. Opin. Struct. Biol.* 22 540–549. 10.1016/j.sbi.2012.06.007 22819665

[B6] Brust H. Orzechowski S. Fettke J. Steup M. (2013). Starch Synthesizing Reactions and Paths: in vitro and in vivo Studies. *J. Appl. Glycosci.* 60 3–20. 10.5458/jag.jag.JAG-2012_018

[B7] Buschiazzo A. Ugalde J. E. Guerin M. E. Shepard W. Ugalde R. A. Alzari P. M. (2004). Crystal structure of glycogen synthase: homologous enzymes catalyze glycogen synthesis and degradation. *EMBO J.* 23 3196–3205. 10.1038/sj.emboj.7600324 15272305 PMC514502

[B8] Cenci U. Nitschke F. Steup M. Minassian B. A. Colleoni C. Ball S. G. (2014). Transition from glycogen to starch metabolism in Archaeplastida. *Trends Plant Sci.* 19 18–28. 10.1016/j.tplants.2013.08.004 24035236

[B9] Chen J. Huang B. Li Y. Du H. Gu Y. Liu H. (2011). Synergistic influence of sucrose and abscisic acid on the genes involved in starch synthesis in maize endosperm. *Carbohydr. Res.* 346 1684–1691. 10.1016/j.carres.2011.05.003 21640984

[B10] Colleoni C. Linka M. Deschamps P. Handford M. G. Dupree P. Weber A. P. (2010). Phylogenetic and biochemical evidence supports the recruitment of an ADP-glucose translocator for the export of photosynthate during plastid endosymbiosis. *Mol. Biol. Evol.* 27 2691–2701. 10.1093/molbev/msq158 20576760

[B11] Coutinho P. M. Deleury E. Davies G. J. Henrissat B. (2003). An evolving hierarchical family classification for glycosyltransferases. *J. Mol. Biol.* 328 307–317. 10.1016/S0022-2836(03)00307-3 12691742

[B12] Crofts N. Abe K. Aihara S. Itoh R. Nakamura Y. Itoh K. (2012). Lack of starch synthase IIIa and high expression of granule-bound starch synthase I synergistically increase the apparent amylose content in rice endosperm. *Plant Sci.* 19 62–69. 10.1016/j.plantsci.2012.05.006 22794919

[B13] Cuesta-Seijo J. A. Nielsen M. M. Marri L. Tanaka H. Beeren S. R. Palcic M. M. (2013). Structure of starch synthase I from barley: insight into regulatory mechanisms of starch synthase activity. *Acta Crystallogr. D. Biol. Crystallogr.* 69 1013–1025. 10.1107/S090744491300440X 23695246

[B14] Dauvillee D. Chochois V. Steup M. Haebel S. Eckermann N. Ritte G. (2006). Plastidial phosphorylase is required for normal starch synthesis in *Chlamydomonas reinhardtii*. *Plant J.* 48 274–285. 10.1111/j.1365-313X.2006.02870.x 17018036

[B15] Delvalle D. Dumez S. Wattebled F. Roldan I. Planchot V. Berbezy P. (2005). Soluble starch synthase I: a major determinant for the synthesis of amylopectin in *Arabidopsis thaliana* leaves. *Plant J.* 43 398–412. 10.1111/j.1365-313X.2005.02462.x 16045475

[B16] Deschamps P. Colleoni C. Nakamura Y. Suzuki E. Putaux J. L. Buleon A. (2008a). Metabolic symbiosis and the birth of the plant kingdom. *Mol. Biol. Evol.* 25 536–548. 10.1093/molbev/msm280 18093994

[B17] Deschamps P. Haferkamp I. D’hulst C. Neuhaus H. E. Ball S. G. (2008b). The relocation of starch metabolism to chloroplasts: when, why and how. *Trends Plant Sci.* 13 574–582. 10.1016/j.tplants.2008.08.009 18824400

[B18] Deschamps P. Moreau H. Worden A. Z. Dauvillee D. Ball S. G. (2008c). Early gene duplication within chloroplastida and its correspondence with relocation of starch metabolism to chloroplasts. *Genetics* 178 2373–2387. 10.1534/genetics.108.087205 18245855 PMC2323822

[B19] Dian W. Jiang H. Wu P. (2005). Evolution and expression analysis of starch synthase III and IV in rice. *J. Exp. Bot.* 56 623–632. 10.1093/jxb/eri065 15642712

[B20] Gamez-Arjona F. M. Li J. Raynaud S. Baroja-Fernandez E. Munoz F. J. Ovecka M. (2011). Enhancing the expression of starch synthase class IV results in increased levels of both transitory and long-term storage starch. *Plant Biotechnol. J.* 9 1049–1060. 10.1111/j.1467-7652.2011.00626.x 21645200

[B21] Gao M. Wanat J. Stinard P. S. James M. G. Myers A. M. (1998). Characterization of dull1, a maize gene coding for a novel starch synthase. *Plant Cell* 10 399–412. 10.2307/3870597 9501113 PMC143999

[B22] Jeon J. S. Ryoo N. Hahn T. R. Walia H. Nakamura Y. (2010). Starch biosynthesis in cereal endosperm. *Plant Physiol. Biochem.* 48 383–392. 10.1016/j.plaphy.2010.03.006 20400324

[B23] Leterrier M. Holappa L. D. Broglie K. E. Beckles D. M. (2008). Cloning, characterisation and comparative analysis of a starch synthase IV gene in wheat: functional and evolutionary implications. *BMC Plant Biol.* 8:98 10.1186/1471-2229-8-98 PMC2576272 18826586

[B24] Momma M. Fujimoto Z. (2012). Interdomain disulfide bridge in the rice granule bound starch synthase I catalytic domain as elucidated by X-ray structure analysis. *Biosci. Biotechnol. Biochem.* 76 1591–1595. 10.1271/bbb.120305 22878205

[B25] Nougue O. Corbi J. Ball S. G. Manicacci D. Tenaillon M. I. (2014). Molecular evolution accompanying functional divergence of duplicated genes along the plant starch biosynthesis pathway. *BMC Evol. Biol.* 14:103 10.1186/1471-2148-14-103 PMC4041918 24884572

[B26] Roldan I. Wattebled F. Mercedes Lucas M. Delvalle D. Planchot V. Jimenez S. (2007). The phenotype of soluble starch synthase IV defective mutants of *Arabidopsis thaliana* suggests a novel function of elongation enzymes in the control of starch granule formation. *Plant J.* 49 492–504. 10.1111/j.1365-313X.2006.02968.x 17217470

[B27] Schnable P. S. Ware D. Fulton R. S. Stein J. C. Wei F. Pasternak S. (2009). The B73 maize genome: complexity, diversity, and dynamics. *Science* 326 1112–1115. 10.1126/science.1178534 19965430

[B28] Schwarte S. Brust H. Steup M. Tiedemann R. (2013). Intraspecific sequence variation and differential expression in starch synthase genes of *Arabidopsis thaliana*. *BMC Res. Notes* 6:84 10.1186/1756-0500-6-84 PMC3608163 23497496

[B29] Sheng F. Jia X. Yep A. Preiss J. Geiger J. H. (2009). The crystal structures of the open and catalytically competent closed conformation of *Escherichia coli* glycogen synthase. *J. Biol. Chem.* 284 17796–17807. 10.1074/jbc.M809804200 19244233 PMC2719418

[B30] Smith A. M. (2008). Prospects for increasing starch and sucrose yields for bioethanol production. *Plant J.* 54 546–558. 10.1111/j.1365-313X.2008.03468.x 18476862

[B31] Sparla F. Falini G. Botticella E. Pirone C. Talame V. Bovina R. (2014). New starch phenotypes produced by TILLING in barley. *PLoS ONE* 9:e107779 10.1371/journal.pone.0107779 PMC4182681 25271438

[B32] Szydlowski N. Ragel P. Raynaud S. Lucas M. M. Roldan I. Montero M. (2009). Starch granule initiation in *Arabidopsis* requires the presence of either class IV or class III starch synthases. *Plant Cell* 21 2443–2457. 10.1105/tpc.109.066522 19666739 PMC2751949

[B33] Wattebled F. Buleon A. Bouchet B. Ral J. P. Lienard L. Delvalle D. (2002). Granule-bound starch synthase I. A major enzyme involved in the biogenesis of B-crystallites in starch granules. *Eur. J. Biochem.* 269 3810–3820. 10.1046/j.1432-1033.2002.03072.x 12153578

[B34] Xu Z. Wang H. (2007). LTR_FINDER: an efficient tool for the prediction of full-length LTR retrotransposons. *Nucleic Acids Res.* 35 W265–W268. 10.1093/nar/gkm286 17485477 PMC1933203

[B35] Yan H. Pan X. Jiang H. Wu G. (2009). Comparison of the starch synthesis genes between maize and rice: copies, chromosome location and expression divergence. *Theor. Appl. Genet.* 119 815–825. 10.1007/s00122-009-1091-5 19593540

[B36] Zeeman S. C. Kossmann J. Smith A. M. (2010). Starch: its metabolism, evolution, and biotechnological modification in plants. *Annu. Rev. Plant Biol.* 61 209–234. 10.1146/annurev-arplant-042809-112301 20192737

[B37] Zhang J. Chen J. Yi Q. Hu Y. Liu H. Liu Y. (2014). Novel role of ZmaNAC36 in co-expression of starch synthetic genes in maize endosperm. *Plant Mol. Biol.* 84 359–369. 10.1007/s11103-013-0153-x 24235061

[B38] Zhang X. Szydlowski N. Delvalle D. D’hulst C. James M. G. Myers A. M. (2008). Overlapping functions of the starch synthases SSII and SSIII in amylopectin biosynthesis in *Arabidopsis*. *BMC Plant Biol.* 8:96 10.1186/1471-2229-8-96 PMC2566982 18811962

