# Supplementary material for: Identification and Phylogenetic Analysis of a Novel Starch Synthase in Maize
Source: Front Plant Sci. 2015 Nov 20;6:1013. doi: 10.3389/fpls.2015.01013 (PMC4653816; doi:10.3389/fpls.2015.01013)

Supplementary Material

Identification and phylogenetic analysis of a novel starch synthase in maize

Hanmei Liu^1^, Guiling Yu^1^, Bin Wei^2^, Yongbin Wang^2^, Junjie Zhang^1^, Yufeng Hu^3^, Yinghong Liu^2^, Guowu Yu^3^, Huaiyu Zhang^1^, Yubi Huang^2,3^*

***Correspondence:** Yubi Huang: [yubihuang@sohu.com](mailto:yubihuang@sohu.com)

# Additional file 1

Table S1. List of sequences used in this study.

| Gene | Nucleotide accession number | Protein accession number | Species name | Prefix of species name |
| --- | --- | --- | --- | --- |
| *GBSS* | JF727262.1 | AEH27527.1 | *Amorphophallus konjac* | Ak |
| *GBSS* | XM_002893700.1 | XP_002893746.1 | *Arabidopsis lyrata* | Al |
| *SSI* | XM_002872057.1 | XP_002872103.1 | *Arabidopsis lyrata* |  |
| *SSIII* | XM_002889845.1 | XP_002889891.1 | *Arabidopsis lyrata* |  |
| *GBSS* | AY149948.1 | AAN31102.1 | *Arabidopsis thaliana* | At |
| *SSI* | NM_122336.4 | NP_197818.1 | *Arabidopsis thaliana* |  |
| *SSII* | NM_110984.2 | NP_186767.1 | *Arabidopsis thaliana* |  |
| *SSIII* | NM_101044.2 | NP_172637.1 | *Arabidopsis thaliana* |  |
| *SSIV* | NM_117934.4 | NP_193558.3 | *Arabidopsis thaliana* |  |
| *SSV* | BT029154 | ABJ17089 | *Arabidopsis thaliana* |  |
| *GBSS* | AF097922.1 | AAC70779.1 | *Astragalus membranaceus* | Am |
| *GBSS* | XM_001696565.1 | XP_001696617.1 | *Chlamydomonas reinhardtii* | Cr |
| *SSII* | XM_001691777.1 | XP_001691829.1 | *Chlamydomonas reinhardtii* |  |
| *SSIII* | XM_001695275.1 | XP_001695327.1 | *Chlamydomonas reinhardtii* |  |
| *SSIV* | XM_001701417.1 | XP_001701469.1 | *Chlamydomonas reinhardtii* |  |
| *SSV* | XM_001701434.1 | XP_001701486.1 | *Chlamydomonas reinhardtii* |  |
| *GBSS* | FJ415205.1 | ACJ11751.1 | *Gossypium hirsutum* | Gh |
| *GBSSI* | FN179380.1 | CAX51363.1 | *Hordeum vulgare* | Hv |
| *SSI* | FN179374.1 | CAX51357.1 | *Hordeum vulgare* |  |
| *SSII* | AY133249.1 | AAN28309.1 | *Hordeum vulgare* |  |
| *SSIIIa* | FN179377.1 | CAX51360.1 | *Hordeum vulgare* |  |
| *SSIIIb* | FN179378.1 | CAX51361.1 | *Hordeum vulgare* |  |
| *SSIV* | FN179379.1 | CAX51362.1 | *Hordeum vulgare* |  |
| *SSII* | AF068834.1 | AAC19119.1 | *Ipomoea batatas* | Ib |
| *GBSS* | EU586115.1 | ACB97677.1 | *Malus x domestica* | Md |
| *GBSS* | X74160.1 | CAA52273.1 | *Manihot esculenta* | Me |
| *SSI* | EF667960.1 | ABV25893.1 | *Manihot esculenta* |  |
| *SSIII* | HQ646361.1 | ADZ30930.1 | *Musa acuminata* | Ma |
| *GBSSI* | AF141954.1 | AAF72561.1 | *Oryza sativa* | Os |
| *GBSSII* | AY069940.1 | AAL58572.1 | *Oryza sativa* |  |
| *SSI* | AY299404.1 | AAP56350.1 | *Oryza sativa* |  |
| *SSIIa* | AB115917.1 | BAD90593.1 | *Oryza sativa* |  |
| *SSIIb* | AF395537.1 | AAK81729.1 | *Oryza sativa* |  |
| *SSIIc* | NM_001071213.1 | NP_001064678.1 | *Oryza sativa* |  |
| *SSIIIa* | AF432915.1 | AAL40942.1 | *Oryza sativa* |  |
| *SSIIIb* | AY100469.1 | AAM49811.1 | *Oryza sativa* |  |
| *SSIVa* | AY373257.1 | AAQ82622.1 | *Oryza sativa* |  |
| *SSIVb* | AY373258.1 | AAQ82623.1 | *Oryza sativa* |  |
| *SSV* | EU621837.1 | ACC78131.1 | *Oryza sativa* |  |
| *GBSS* | XM_003079885.1 | XP_003079933.1 | *Ostreococcus tauri* | Ot |
| *SSIII* | XM_003082548.1 | XP_003082596.1 | *Ostreococcus tauri* |  |
| *GBSS* | AB029546.1 | BAA82346.1 | *Phaseolus vulgaris* | Pv |
| *SSIII* | AB293998.1 | BAF49176.1 | *Phaseolus vulgaris* |  |
| *SSI* | XM_001784372.1 | XP_001784424.1 | *Physcomitrella patens* | Pp |
| *SSII* | XM_001778166.1 | XP_001778218.1 | *Physcomitrella patens* |  |
| *SSIV* | XM_001781369.1 | XP_001781421.1 | *Physcomitrella patens* |  |
| *SSV* | XM_001758862.1 | XP_001758914 | *Physcomitrella patens* |  |
| *GBSS* | AJ345045.1 | CAC69955.1 | *Pisum sativum* | Ps |
| *SSII* | X88790.1 | CAA61269.1 | *Pisum sativum* |  |
| *SSII* | XM_002324025.1 | XP_002324061.1 | *Populus trichocarpa* | Pt |
| *SSIII* | XM_002305535.1 | XP_002305571.1 | *Populus trichocarpa* |  |
| *SSV* | XM_002310280.2 | XP_002310316.1 | *Populus trichocarpa* |  |
| *GS* | glgA (A9601_06651) | YP_001009058.1 | *Prochlorococcus marinus AS9601* | Pm |
| *SSIII* | XM_002518430.1 | XP_002518476.1 | *Ricinus communis* | Rc |
| *GBSSI* | AB089141.1 | BAC06486.1 | *Setaria italica* | Si |
| *SSI* | Y10416.1 | CAA71442.1 | *Solanum tuberosum* | St |
| *SSV* | NM_001288111.1 | NP_001275040.1 | *Solanum tuberosum* |  |
| *SSIII* | GQ221265.1 | ACT09059.1 | *Solanum lycopersicum* | Sl |
| *GBSSI* | U23945.1 | AAC49804.1 | *Sorghum bicolor* | Sb |
| *GBSSII* | XM_002461844.1 | XP_002461889.1 | *Sorghum bicolor* |  |
| *SSIIIa* | EU620720.1 | ACC86846.1 | *Sorghum bicolor* |  |
| *SSIIIb* | EU620721.1 | ACC86847.1 | *Sorghum bicolor* |  |
| *SSIV* | XM_002440083.1 | XP_002440128.1 | *Sorghum bicolor* |  |
| *SSV* | KP192926 | KP192926 | *Sorghum bicolor* |  |
| *GS* | glgA (GSYN1639) | YP_730734.1 | *Synechoccus sp. CC9311* | Ss |
| *GBSSI* | AY050174.1 | AAL05405.1 | *Triticum aestivum* | Ta |
| *GBSSII* | AF109395.1 | AAF14233.1 | *Triticum aestivum* |  |
| *SSI* | AF091803.1 | AAD54661.1 | *Triticum aestivum* |  |
| *SSII* | AF155217.2 | AAD53263.1 | *Triticum aestivum* |  |
| *SSIIIa* | AF258608.1 | AAF87999.1 | *Triticum aestivum* |  |
| *SSIIIb* | EU333946.2 | ABY56823.2 | *Triticum aestivum* |  |
| *SSIV* | AY044844.1 | AAK97773.1 | *Triticum aestivum* |  |
| *SSII* | FJ561746.1 | ACL98485.1 | *Vigna radiata* | Vr |
| *SSII* | FJ561745.1 | ACL98484.1 | *Vigna unguiculata* | Vu |
| *SSIII* | AJ225088.1 | CAB40374.1 | *Vigna unguiculata* |  |
| *SSIV* | AJ006752.1 | CAB40375.1 | *Vigna unguiculata* |  |
| *SSII* | XM_002278434.1 | XP_002278470.1 | *Vitis vinifera* | Vv |
| *GBSSI* | X03935.1 | CAA27574.1 | *Zea mays* | Zm |
| *GBSSII* | EF472248.1 | NP_001106039 | *Zea mays* |  |
| *SSI* | AF036891.2 | AAB99957.2 | *Zea mays* |  |
| *SSIIa* | AF019296.1 | AAD13341.1 | *Zea mays* |  |
| *SSIIb* | AF019297.1 | AAD13342.1 | *Zea mays* |  |
| *SSIIc* | NM_001114648.1 | NP_001108120.1 | *Zea mays* |  |
| *SSIIIa* | NM_001111411.1 | NP_001104881.1 | *Zea mays* |  |
| *SSIIIb* | NM_001112545.1 | NP_001106015.1 | *Zea mays* |  |
| *SSIV* | EU599036.1 | ACC63897.1 | *Zea mays* |  |
| *SSV* | NM_001130131.1 | NP_001123603.1 | *Zea mays* |  |
| SSVa | XM_006572919.1 | XP_006572982.1 | Glycine max | Gm |
| SSVb | GLYMA09G33665(Gramene) |  | Glycine max |  |

For each sequence, we showed the “Gene name”, the “Nucleotide/Protein Accession number” in NCBI and the “Species name”. The prefix of each gene was labeled with the initials of the genus and species.

# Additional file 2


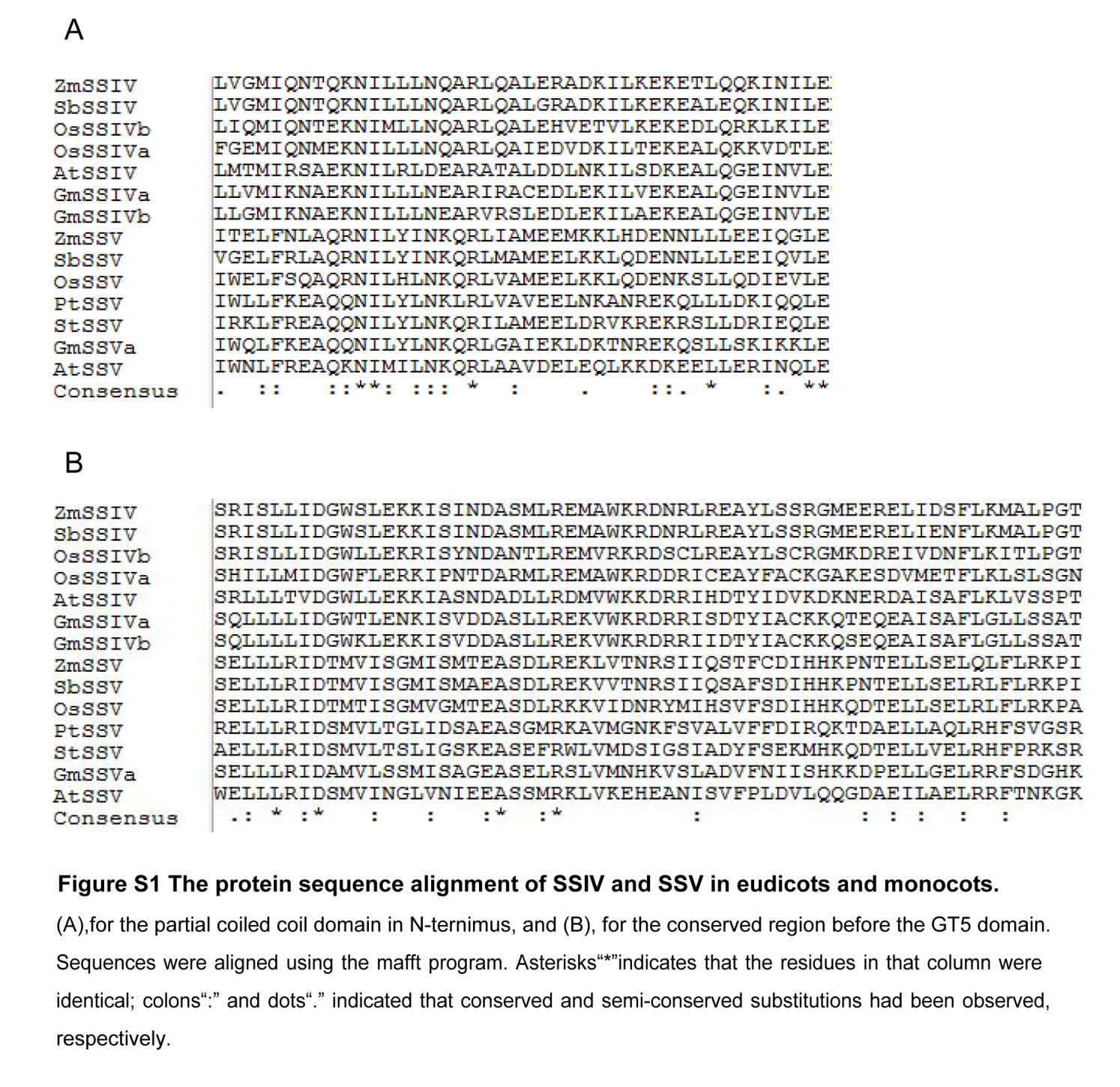

Supplement: Supplementary file 1 [file Table_1.DOCX]
